# Development of a monoclonal antibody specific to the endonuclease domain of the human LINE-1 ORF2 protein

**DOI:** 10.1186/s13100-014-0029-x

**Published:** 2014-12-10

**Authors:** Mark Sokolowski, Cecily B DeFreece, Geraldine Servant, Kristine J Kines, Dawn L deHaro, Victoria P Belancio

**Affiliations:** Department of Structural and Cellular Biology, Tulane School of Medicine, Tulane Cancer Center, and Tulane Center for Aging, New Orleans, LA 70112 USA; Department of Biology, Xavier University, 1 Drexel Drive, Box 85, New Orleans, LA 70125-7918 USA; Department of Epidemiology, Tulane School of Public Health, Tulane Cancer Center, New Orleans, LA 70112 USA

**Keywords:** Endonuclease, *In vitro* assay, L1, L1 antibody, LINE-1, ORF2, Retrotransposition

## Abstract

**Background:**

LINE-1 (L1) retrotransposons are common occupants of mammalian genomes representing about a fifth of the genetic content. Ongoing L1 retrotransposition in the germ line and somatic tissues has contributed to structural genomic variations and disease-causing mutations in the human genome. L1 mobilization relies on the function of two, self-encoded proteins, ORF1 and ORF2. The ORF2 protein contains two characterized domains: endonuclease and reverse transcriptase.

**Results:**

Using a bacterially purified endonuclease domain of the human L1 ORF2 protein, we have generated a monoclonal antibody specific to the human ORF2 protein. We determined that the epitope recognized by this monoclonal antibody includes amino acid 205, which is required for the function of the L1 ORF2 protein endonuclease. Using an *in vitro* L1 cleavage assay, we demonstrate that the monoclonal anti-ORF2 protein antibody partially inhibits L1 endonuclease activity without having any effect on the *in vitro* activity of the human AP endonuclease.

**Conclusions:**

Overall, our data demonstrate that this anti-ORF2 protein monoclonal antibody is a useful tool for human L1-related studies and that it provides a rationale for the development of antibody-based inhibitors of L1-induced damage.

**Electronic supplementary material:**

The online version of this article (doi:10.1186/s13100-014-0029-x) contains supplementary material, which is available to authorized users.

## Background

**Figure 1:**
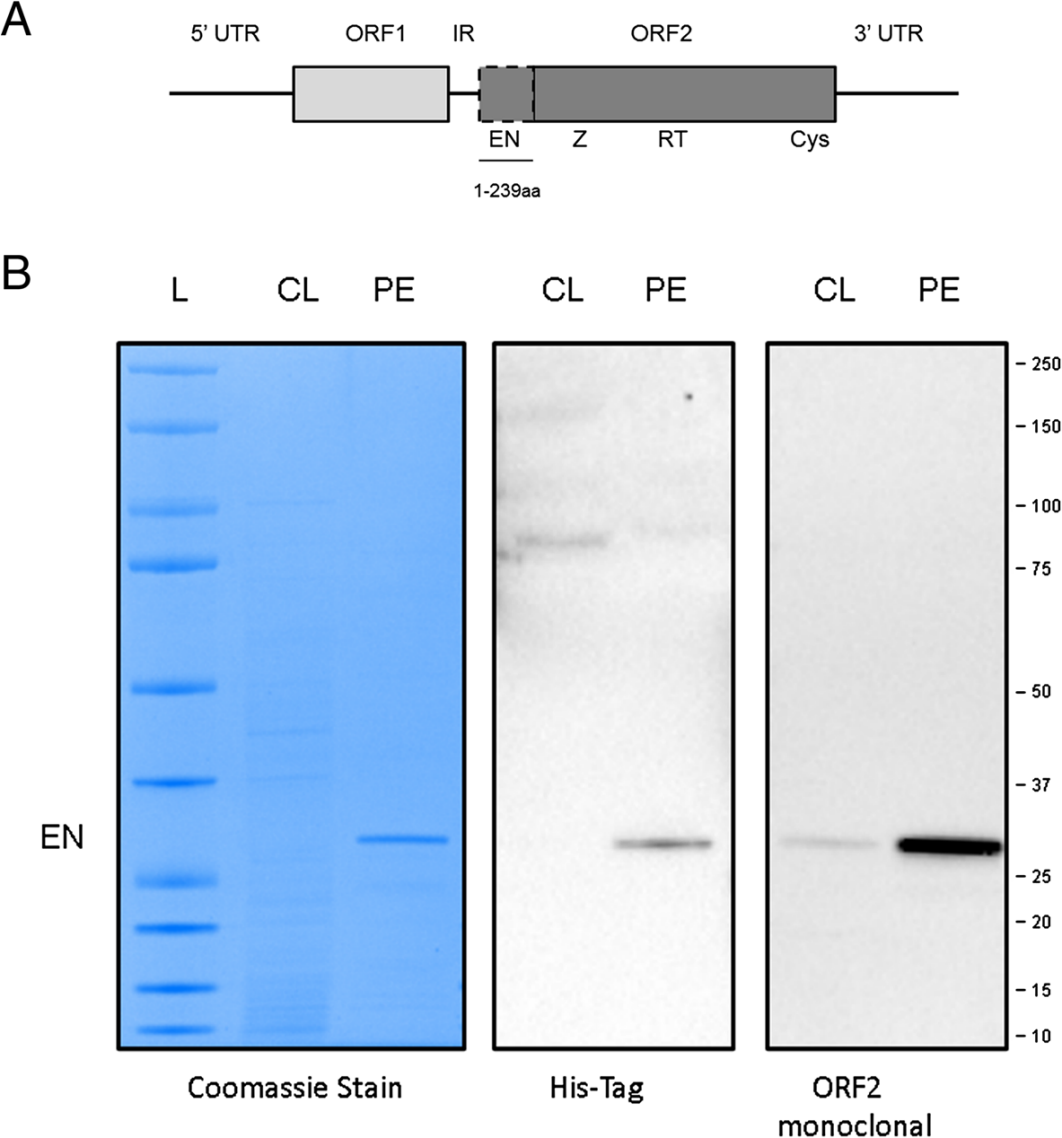
**Analysis of bacterially purified human endonuclease. (A)** Schematic of a full-length L1, which contains a 5′ untranslated region (UTR) followed by an ORF1 sequence, an intergenic region, an ORF2 sequence, and a 3′ UTR. The EN region of the ORF2 sequence subcloned to generate the purified ORF2p endonuclease (EN, 1-239aa) is indicated with a dashed box. **(B)** **(Left panel)** Coomassie stain of SDS-PAGE gel. Ladder (L), clarified lysate from bacteria expressing ORF2p endonuclease (CL), and final purified elution (PE) are shown; 500 ng of protein was loaded in each lane. **(Middle panel)** Western blot analysis of 500 ng of CL and PE with HIS-tag specific antibodies. The ORF2p endonuclease used in this study has a HIS-tag fused to its N-terminus (expected size of the His EN protein is 29 kilodaltons, kDa). **(Right panel)** Western blot analysis of 500 ng of CL and PE with a custom anti-human ORF2p monoclonal antibody. Molecular markers on the right, 10 to 250 kDa.

Long interspersed element-1 (L1) is an autonomous non-long terminal repeat retrotransposon that has parasitized the human genome for millions of years. L1 has shaped the evolution of the human genome through a copy-and-paste mobilization of itself [1], as well as the short interspersed element (SINE) Alu [2], SINE-VNTR-Alu elements (SVA) [3], and processed cellular transcripts [4]. Functional full-length L1 transcripts contain two open reading frames (ORFs) encoding ORF1 and ORF2 proteins (ORF1p and ORF2p, respectively) (Figure 1A). These L1 proteins exhibit *cis*-preference for their encoding L1 mRNA [5-7] and are utilized in *trans* by the Alu and SVA elements [2,3,8]. L1, Alu, and SVA form ribonucleoprotein (RNP) particles which reach the nucleus to complete their replication cycles by integrating in the host genome via a process of target-primed reverse transcription [9,10]. This copy-and-paste process has produced approximately 500,000 L1 loci, accounting for about 17% of the human genome, and over 1,000,000 copies of Alu, which comprise about 11% of our genome [11]. The majority of the L1 loci are 5′ truncated with about 80 to 100 full-length L1 copies demonstrated to be retrotranspositionally active [12-16].

L1 proteins are produced from the full-length L1 mRNA with significantly different efficiencies, mostly owing to the unconventional translation from the bicistronic L1 mRNA [17-20] (Figure 1A). Detection of both L1-encoded proteins is important in understanding L1 biology since they play critical, but different roles in the L1 replication cycle. The human ORF2p is a 149 kilodalton (kDa) protein with three annotated domains: an N-terminal endonuclease (EN) domain [21], a reverse transcriptase (RT) domain [22], and a C-terminal domain [23] with putative RNA binding activity [24]. Human and mouse L1 ORF2 proteins exhibit a high degree of sequence homology and conservation of function making findings in mouse model systems biologically relevant to the replication cycle of the human L1 [25,26]. Although much has been learned about ORF2p function *in vitro* and in mammalian cells using overexpressed tagged ORF2 proteins and polyclonal anti-ORF2p antibodies [27-30], having a monoclonal antibody that can detect the untagged human ORF2 protein would be a useful molecular tool to study the requirements for the human L1 ORF2p expression and activity. It would also aid in advancing our appreciation of the ORF2p impact on host genome stability and in understanding the consequences of its activity to human health.

To satisfy the need for a continuous source of antibodies to detect L1 ORF2p, we developed an anti-ORF2p monoclonal antibody capable of recognizing sequences within the endonuclease domain of the human ORF2 protein. This monoclonal antibody is specific to the human ORF2p and can detect the full-length ORF2 protein, as well as truncated ORF2 proteins overexpressed in mammalian cells. Using a recombinant human L1 endonuclease purified from bacterial cells as a standard [31,32], we determined the sensitivity of this monoclonal anti-human ORF2p antibody. The unique location of the epitope, encompassing a position required for the function of the human endonuclease domain, allowed us to test the ability of this monoclonal anti-ORF2p antibody to inhibit L1 endonuclease activity *in vitro* using a fluorescence-based cleavage assay.

## Results

### Generation of monoclonal antibody against human L1 ORF2p endonuclease

A recombinant human protein containing an ORF2p EN domain N-terminally fused to a His-tag was purified from bacterial cells as previously described [31-33], subjected to SDS-PAGE, and visualized using Coomassie stain (Figure 1B, Coomassie panel, expected product of 29 kDa). The efficiency of purification was also confirmed using antibodies against the His-tag fused to the N-terminus of the ORF2p EN (Figure 1B, His-Tag panel). This purified recombinant human EN protein was used for the immunization of Balb/c mice to generate monoclonal anti-ORF2p antibodies following a standard immunization protocol (see Methods). This approach resulted in a positive hybridoma clone which was used to produce the purified anti-ORF2p monoclonal antibodies. Western blot analysis using this custom ORF2p monoclonal antibody detected a product of the expected size in the clarified lysate and the final elution of the human EN protein used for inoculation (Figure 1B, ORF2 monoclonal panel).

### Anti-ORF2p monoclonal antibody is specific to the ORF2 protein of human origin

**Figure 2:**
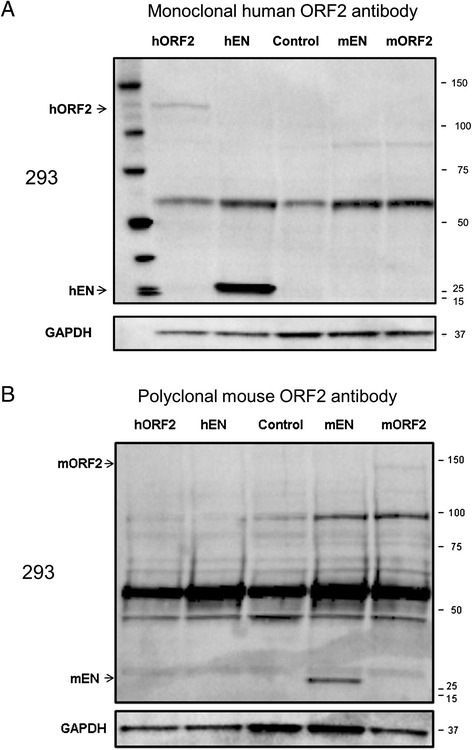
**Analysis of specificity of the custom anti-human ORF2p monoclonal antibody in human cells. (A)** Western blot analysis of mouse and human ORF2 (predicted size 150 and 149 kDa, respectively) and EN (predicted size 30 and 26 kDa, respectively) proteins generated from expression plasmids containing codon-optimized human ORF2 (hORF2), a codon-optimized sequence corresponding to the human ORF2 endonuclease fragment (hEN), codon-optimized mouse ORF2 (mORF2), and a codon-optimized sequence corresponding to the mouse ORF2 endonuclease fragment (mEN) transiently transfected in 293 cells. Custom anti-human ORF2p monoclonal antibody specifically detects proteins of human origin. **(B)** Western blot analysis of the same samples as in A was performed with custom anti-mouse ORF2p polyclonal antibodies which specifically detect proteins of mouse origin. Control lane indicates cells transiently transfected with an empty vector. GAPDH is used as a loading control; 15 to 150 kDa on the right indicate positions of molecular markers.

We determined that our anti-ORF2p monoclonal antibody detects full-length ORF2p and ORF2p endonuclease in total cell lysates from 293 cells transiently transfected with plasmids containing human codon-optimized full-length ORF2 or the ORF2 endonuclease sequences (Figure 2A, lane hORF2 and hEN). Because the endonuclease domain of the L1 ORF2 protein is highly conserved between the human and mouse ORF2 proteins, we tested whether our antibody discriminates between ORF2 proteins of human and mouse origin. Plasmids encoding mouse codon-optimized full-length ORF2 or ORF2 endonuclease sequences were transiently transfected into 293 cells and total cellular lysates were analyzed by SDS-PAGE followed by immunoblotting with the anti-ORF2p monoclonal antibody. This approach determined that the anti-ORF2p monoclonal antibody does not detect mouse ORF2 or EN proteins (mORF2p and mENp, respectively) even though it detected both human ENp and ORF2p (Figure 2A, monoclonal Ab panel). The mouse ENp and ORF2p were detected when Western blot analysis was performed with polyclonal antibodies raised against the endonuclease domain of the mouse ORF2p [28] (Figure 2B, mouse Ab panel) confirming that the proteins are expressed under these transfection conditions.

### Anti-ORF2p monoclonal antibody recognizes an epitope which includes amino acid 205 of the human ORF2p endonuclease

**Figure 3:**
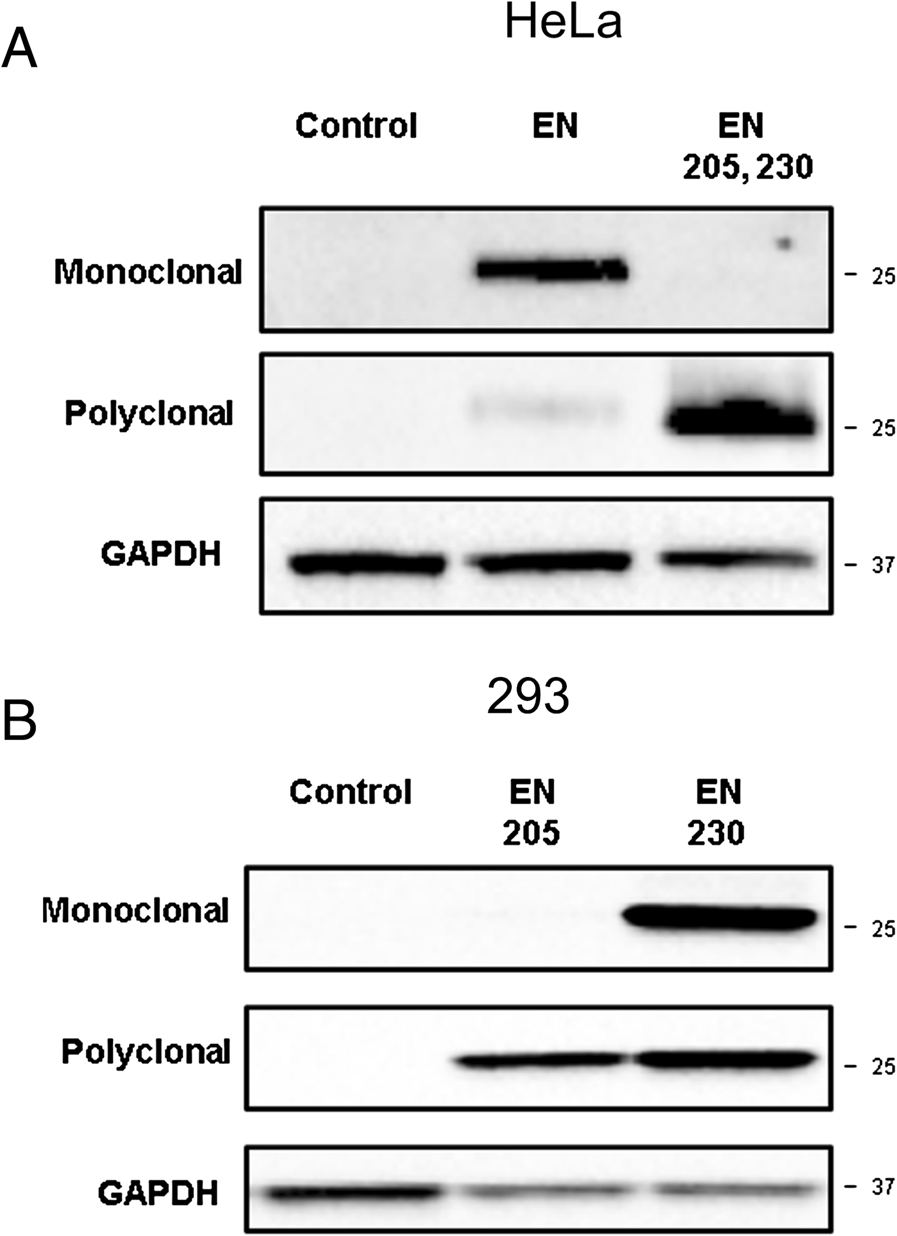
**Analysis of expression of functional and non-functional human ORF2 protein in human cells. (A)** Western blot analysis of proteins generated from expression plasmids containing codon-optimized, functional ORF2 endonuclease sequence (EN) and non-functional ORF2 endonuclease sequence transiently transfected in HeLa cells. Western blot analysis is performed with anti-human ORF2p monoclonal antibody **(top)**, the previously described anti-human ORF2p endonuclease polyclonal antibodies **(middle)**, and GAPDH antibodies **(bottom)**. The non-functional ORF2 endonuclease sequence (EN 205, 230) has mutations resulting in expression of inactive ENp with D205A and H230A mutations. **(B)** The same experiment and analysis as in A, but using 293 cells. Western blot analysis of codon-optimized, non-functional ORF2 endonuclease sequences containing single inactivating mutations D205A or H230A (EN205 and EN 230, respectively) transiently transfected in 293 cells with anti-human ORF2 monoclonal antibody **(top)** or previously described anti-human ORF2p endonuclease polyclonal antibodies **(middle)**. Expected EN protein size is 26 kDa. Control lane indicates cells transiently transfected with an empty vector; 25 and 37 kDa on the right indicate positions of molecular markers.

Many experimental approaches designed to analyze the expression and function of ORF2p involve the use of both functional and non-functional ORF2 proteins. The most commonly used mutations, which abolish the activity of the ORF2p endonuclease, are D205A and H230A [21,34]. Western blot analysis of total cellular lysates from human and mouse cells transiently transfected with EN or EN 205, 230 plasmids containing codon-optimized sequences producing functional or non-functional (D205A, H230A double mutant) human endonucleases demonstrated that the anti-ORF2p monoclonal antibody detects the active, but not the mutated, endonuclease protein (Figure 3A, monoclonal; Additional file 1: Figure S1, monoclonal). Both proteins were detected using the polyclonal anti-ORF2p antibody [28] (Figure 3A; Additional file 1: Figure S1).

**Figure 4:**
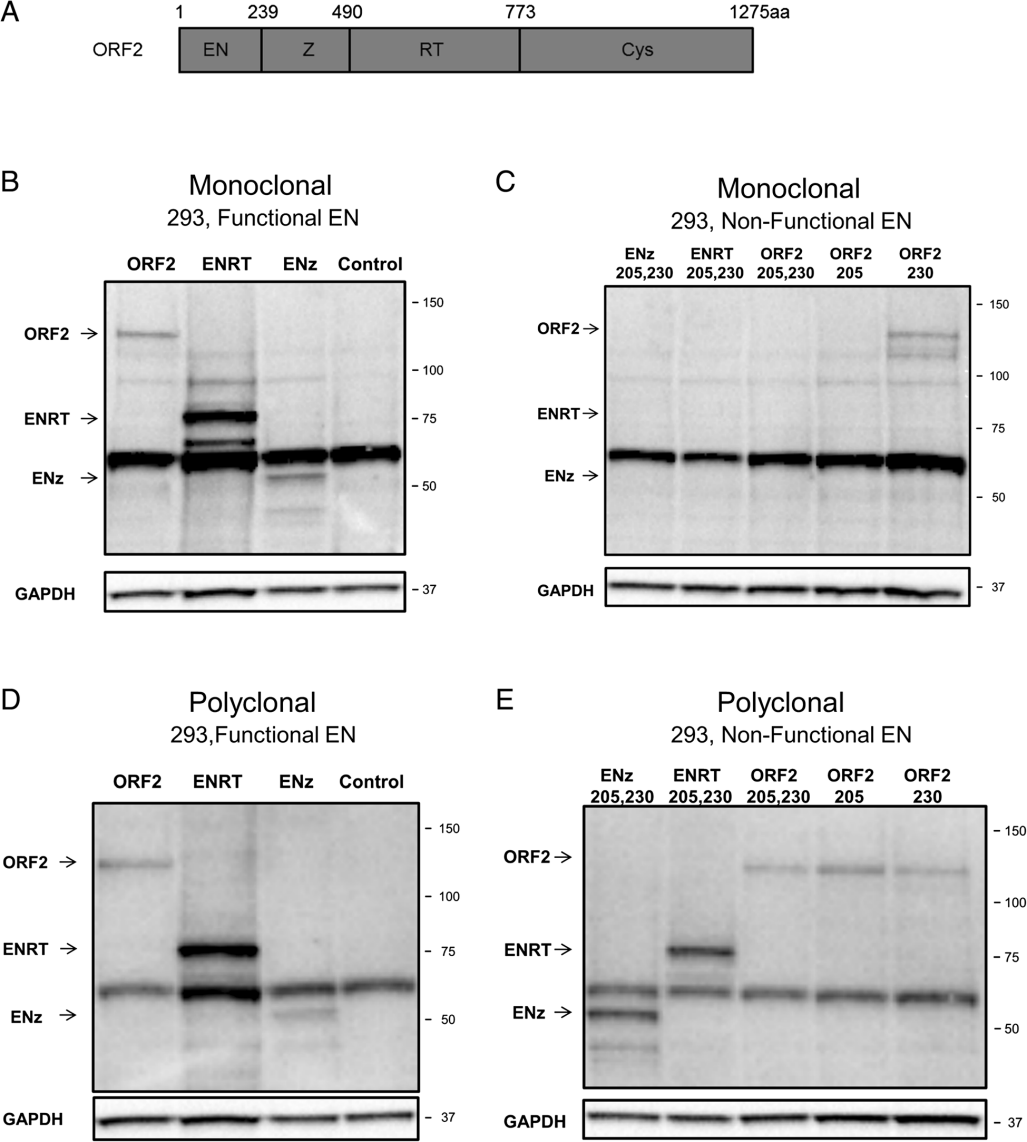
**Analysis of expression of functional and non-functional full-length and truncated human ORF2 proteins in human cells. (A)** Schematic of L1 ORF2 protein. The following ORF2p domains are listed: Endonuclease domain (EN), Z domain (Z), Reverse Transcriptase domain (RT), and the Cysteine-Rich domain (Cys). Amino acid boundaries of each domain are listed. **(B)** Western blot analysis of the full-length and truncated ORF2 proteins generated from expression plasmids containing codon-optimized, functional full-length ORF2 (ORF2) and C-terminally truncated ORF2 sequences transiently transfected in 293 cells with monoclonal antibody. Control lane indicates cells transiently transfected with an empty vector. **(C)** Western blot analysis of proteins with two inactivating mutations are labeled as 205, 230, single mutants are labeled as 205 or 230 generated from expression plasmids containing codon-optimized, mutant of the constructs described in A transiently transfected in 293 cells with monoclonal antibody. Constructs containing two inactivating mutations are labeled as 205, 230, single mutants are labeled as 205 or 230. **(D)** Western blot analysis of the same samples described in A with polyclonal anti-ORF2p antibodies. **(E)** Western blot analysis of the same samples described in B with anti-human ORF2p polyclonal antibodies. GAPDH is used as a loading control; 50 to 150 kDa and 37 kDa on the right indicate molecular markers. Arrows denote bands of expected molecular weights for each construct listed.

To determine which EN mutation is responsible for the loss of detection by our anti-ORF2p monoclonal antibody, total cellular lysates from cells transiently transfected with EN 205 and EN 230 plasmids expressing non-functional endonucleases with D205A or H230A mutations were used for Western blot analysis with the anti-ORF2p monoclonal antibody (Figure 3B, monoclonal). This approach demonstrated that the anti-ORF2p monoclonal antibody detects ENp containing the H230A mutation, but not the ENp with the D205A mutation (Figure 3B, monoclonal). Both ENp mutants are readily detected with the anti-ORF2p polyclonal antibody [27,28], demonstrating that both proteins are produced under these transfection conditions (Figure 3B, polyclonal). A similar result was obtained when the monoclonal anti-ORF2p antibody was used to detect transiently expressed functional (ORF2) and non-functional (single and double mutants) full-length human ORF2 proteins (ORF2 205, ORF2 230, and ORF2 205,230, respectively) as well as truncated, functional, and double mutant human ORF2 proteins (ENz and ENRT) [28] (Figure 4A–E, ORF2, ENz, and ENRT). The anti-ORF2p monoclonal antibody specifically detected functional, but not the non-functional ENz, ENRT, and ORF2 proteins containing the D205A and H230A mutations, even though all of these proteins were produced in these cells as confirmed by Western blot analysis using polyclonal anti-ORF2p antibodies (Figure 4D,E). These results support that the epitope recognized by the anti-ORF2p monoclonal antibody includes amino acid 205 of the human ORF2p endonuclease domain.

### Sensitivity of the anti-ORF2p monoclonal antibody

**Figure 5:**
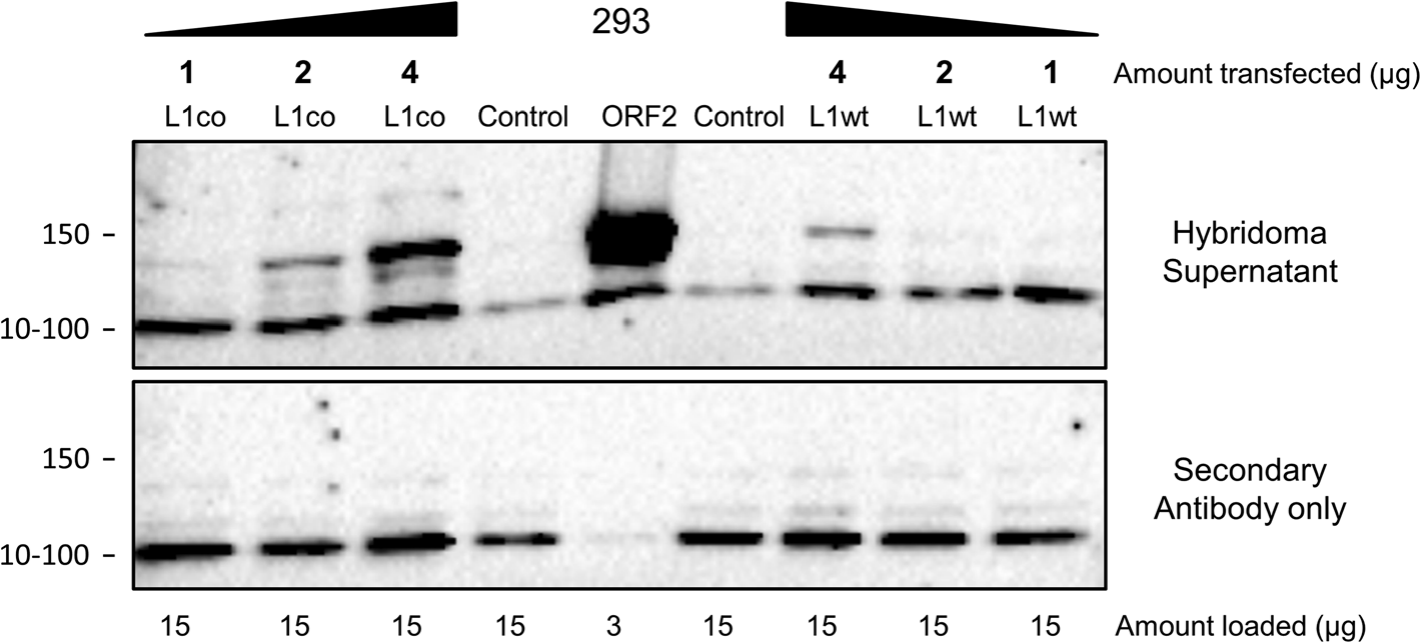
**Analysis of ORF2p generated from a functional wild-type and a functional codon-optimized full-length L1 expression plasmids in 293 cells.** **(Top)** Western blot analysis of ORF2p generated from expression plasmids containing a full-length wild-type L1 (L1wt), a full-length codon-optimized L1 (L1co), or a codon-optimized ORF2 transiently transfected in 293 cells with supernatant collected from cultured hybridoma cells producing anti-ORF2 antibody; 293 cells were transfected with 1, 2, or 4 μg of the L1wt or L1co expression plasmids or 2 μg of the ORF2 expression plasmid and total protein was harvested 24 hours after transfection. Control lane indicates cells transiently transfected with an empty vector. Positions of molecular markers are indicated on the right as 100 or 150 kDa. **(Bottom)** The same experiment and analysis as in **(top)**, but using secondary antibodies only. Positions of molecular markers are indicated on the right as 100 or 150 kDa. Total amount of 293 cell lysate loaded is in μg.

**Figure 6:**
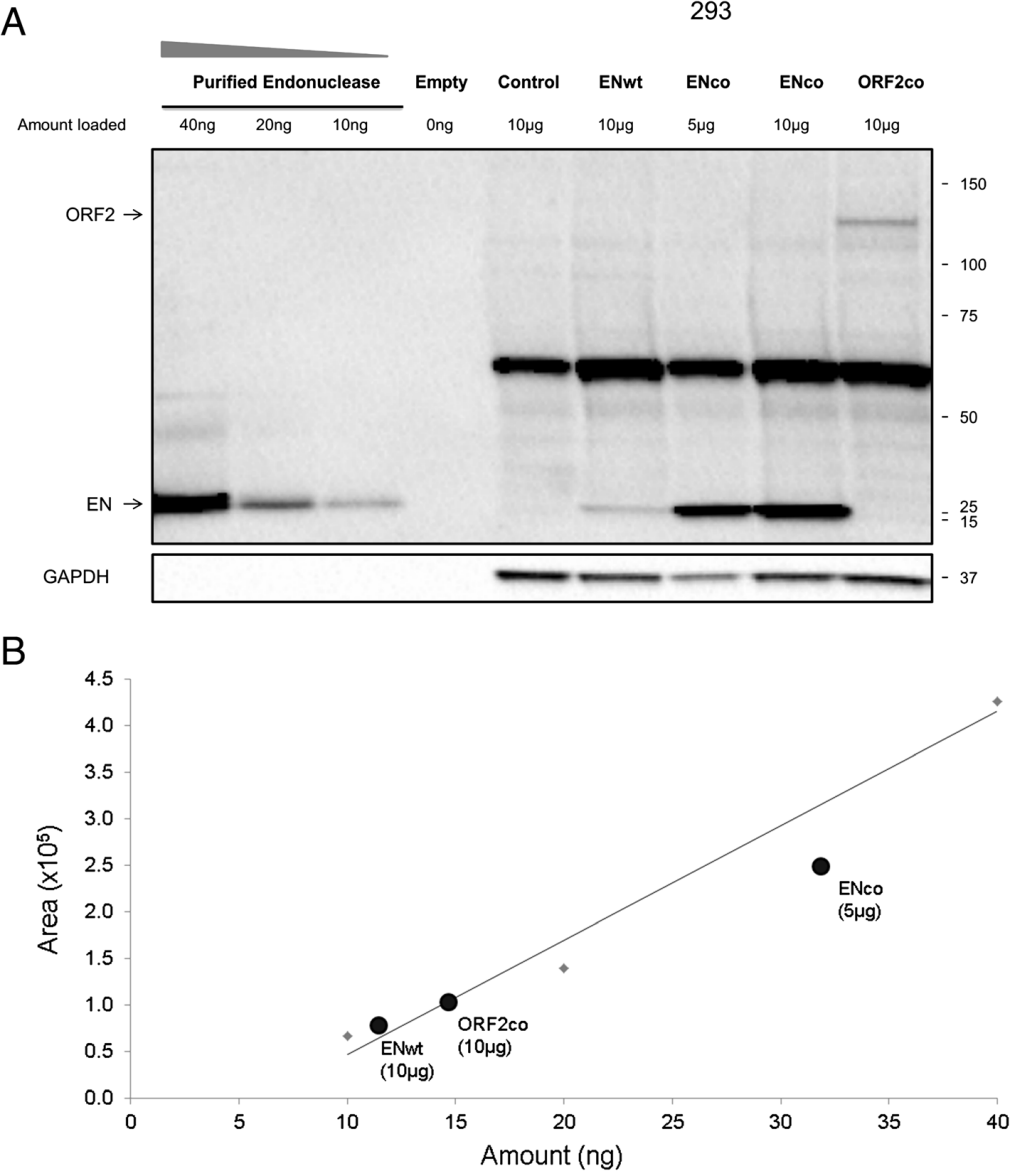
**Analysis of sensitivity of the custom anti-human ORF2p monoclonal antibody. (A)** Western blot analysis of protein generated from expression plasmids containing wild-type ORF2 endonuclease sequence (ENwt), codon-optimized ORF2 endonuclease sequence (ENco), and codon-optimized ORF2 sequence (ORF2) transiently transfected in 293 cells with our monoclonal antibody; 5 or 10 μg of the whole cell lysate was used for analysis as indicated. Control lane indicates cells transiently transfected with an empty vector. Bacterially purified endonuclease was loaded at 0 (empty, buffer only), 10, 20, and 40 ng. GAPDH is used as a loading control; 15 to 150 kDa on the right indicate positions of molecular markers. Arrows denote bands of expected sizes for each protein. **(B)** A standard curve was generated using the quantitation of the increasing amounts of the bacterially purified endonuclease shown in **A**. Signals detected for ORF2co, ENco, and ENwt are plotted and labeled with the respective names of the proteins.

The advent of L1 expression plasmids containing codon-optimized sequences greatly facilitated our ability to detect L1-encoded proteins in transfected mammalian cells [35,36]. However, it remains important to study L1 proteins generated from wild-type L1 sequences and to understand the difference in expression levels between proteins generated from codon-optimized and wild-type L1 sequences. As with the wild-type full-length L1 and ORF2 expression plasmids, codon-optimized full-length L1 expression plasmids produce much less ORF2 protein than those containing a codon-optimized ORF2 sequence [20,25]. Consistent with this fact, our anti-ORF2p monoclonal antibody detected different levels of ORF2p in cells transfected under the same conditions with L1 expression plasmids containing wild-type or codon-optimized sequences [6,36] (Figure 5). Transient transfection of 293 cells with plasmids containing codon-optimized ORF2 or full-length wild-type L1 sequences produced the highest and the lowest levels of ORF2 protein, respectively. Transfection of increasing amounts of plasmids containing codon-optimized or wild type full-length L1 sequences demonstrated that detectable levels of ORF2p were observed when 2 and 4 μg of the respective plasmids were used. No signal consistent with detection of endogenous ORF2p in 293, Ntera2, nor HeLa cells was observed (Additional file 2: Figure S2). Using the recombinant human endonuclease purified from bacterial cells as a standard, we determined that 27.6 μg of our anti-ORF2p monoclonal antibody is able to detect 10 ng (2.2 × 10^17^ molecules) of the purified hEN under these blotting conditions (Figure 6A). Based on the standard curve generated by Western blot analysis of the recombinant human EN purified from bacterial cells, we determined that transfection of expression plasmids containing codon-optimized human L1 sequences produce 5 to 6 times more endonuclease protein than that observed from cells transfected with equivalent amounts of plasmids containing wild-type sequences (Figure 6, ENwt and ENco).

### Monoclonal anti-ORF2p antibody inhibits L1 endonuclease activity in an *in vitro* endonuclease cleavage assay

**Figure 7:**
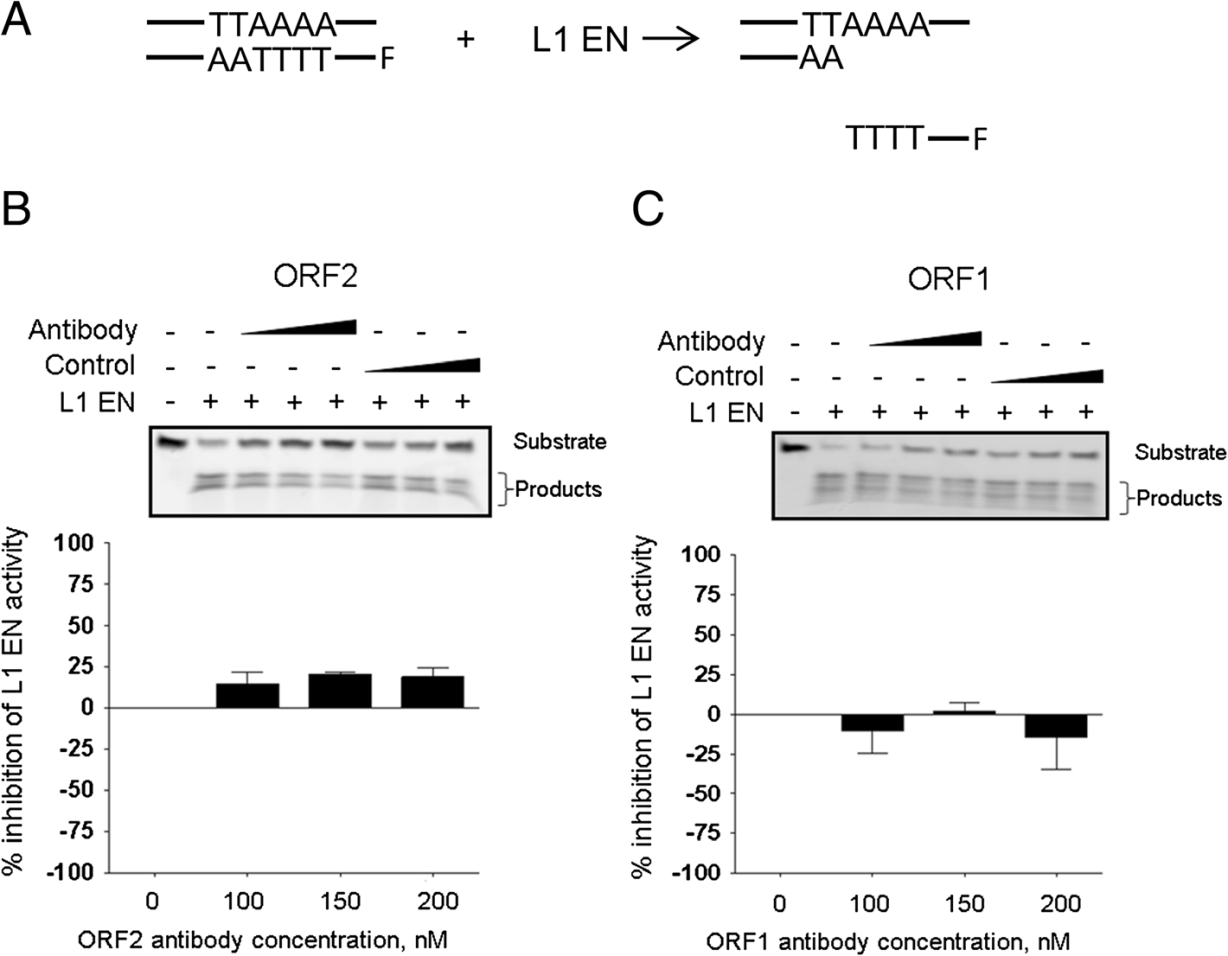
**Monoclonal anti-ORF2p antibody inhibits L1 endonuclease activity in an** ***in vitro*** **endonuclease cleavage assay. (A)** Schematic of *in vitro* endonuclease cleavage assay. Double-stranded DNA containing L1 ORF2 endonuclease consensus target sequence with 5′ tagged with fluorophore. L1 ORF2 endonuclease is added, DNA is cleaved releasing the fluorphore, which can be quantitated. **(B)** **(Top)** SDS-PAGE analysis of the products resulting from the *in vitro* endonuclease assay with or without the addition of the monoclonal anti-ORF2 antibody (0, 100, 150, 200 nM). Antibody (ORF2) denotes the addition of the monoclonal anti-ORF2 antibody, control indicates the addition of the same volume of the buffer used for the reactions containing monoclonal anti-ORF2p antibody, and L1 EN denotes bacterially purified human ORF2 endonuclease. **(Bottom)** Quantitation of the results of the *in vitro* endonuclease cleavage assay in A (see Methods). Results were normalized to 0 nM control (n = 3). **(C)** Same experimental approach as in **B**, but anti-hORF1p antibody was added to the *in vitro* endonuclease cleavage assay.

The unique epitope location within the L1 EN as well as the antibody’s ability to detect natively folded ORF2p endonuclease purified from bacterial cells (Additional file 3: Figure S3) open a possibility that the monoclonal antibody may inhibit L1 endonuclease activity. For this purpose, a previously reported *in vitro* endonuclease cleavage assay was used to measure L1 EN activity [31,32]. Figure 7A shows a schematic of the DNA products expected to be observed upon cleavage of the substrate DNA by the L1 EN at the L1 EN site present in the template DNA sequence. Figure 7B demonstrates detection of the expected cleavage products resolved by PAGE when a bacterially purified, functional human L1 EN protein is present in the reaction. The addition of increasing amounts of the monoclonal anti-ORF2p antibody resulted in about 25% reduction of the cleaved products (Figure 7B). This effect was not observed when an unrelated, anti-ORF1p antibody was included in the reaction (Figure 7C). A similar *in vitro* endonuclease cleavage assay using a functionally-related recombinant human apurinic/apyrimidinic endonuclease 1 (APE 1), which shares sequence homology with the L1-encoded endonuclease, was used to test the specificity of this effect. We observed no change in the APE 1 activity upon the addition of the highest amount of the L1 EN-specific antibody (200 nM; Additional file 4: Figure S4).

## Discussion

L1 is responsible for all of the retrotransposon-induced genomic instability in the human genome, as it is the only active source of the functional ORF1 and ORF2 proteins required for mobilization of LINEs, SINEs, and SVA elements [37-42]. L1 expression and retrotransposition are suppressed by many diverse cellular pathways in order to minimize the genomic damage inflicted by L1 activity [20,43-50]. L1 encodes an ORF2 protein with several identified functions essential for the retrotransposition process. These include the endonuclease [21] and reverse transcriptase [22] activities, and a putative RNA binding domain within the C-terminus of the protein [24]. Studies geared toward understanding the biological relevance of this multifunctional protein and its effect on human health necessitate manipulations involving changes in protein sequence as well as characterization of the expression of resulting ORF2p variants *in vitro*, in cultured cells, and *in vivo*. To satisfy this requirement, polyclonal antibodies against the L1 ORF2 protein of mouse and human origin have been previously reported [27-29].

We have developed a custom monoclonal antibody to the human L1 ORF2p endonuclease domain that will help to advance future studies involving ORF2p expression and function. The monoclonal nature of the antibody provides a continuous source of antibody, thereby eliminating the inherent issue with reproducibility commonly associated with different batches of polyclonal antibodies raised against the same antigen. Similar to previously reported polyclonal antibodies [27], our monoclonal anti-ORF2p antibody detects untagged ORF2 protein expressed from the plasmids containing full-length wild-type or codon-optimized L1 elements. This characteristic is beneficial because the addition of different tags can interfere with L1 protein function or subcellular localization [20,51]. Using bacterially purified endonuclease protein, we generated a standard curve that allowed us to determine the sensitivity of our monoclonal antibodies, which is about 10 ng of the purified protein under the described detection conditions (Figure 6). Consistent with the previous reports, we confirmed that codon-optimization of the human L1 ORF2 sequence results in a 5- to 6-fold increase in EN protein production [35,36]. As we were unable to detect endogenously expressed L1 ORF2p in HeLa and 293 cells, our calculations suggest that endogenous levels of L1 ORF2p expression are less than 10 ng of protein per 10 μg of cellular lysate.

Additionally, we determined that our antibody specifically recognizes human, but not mouse, ORF2 protein despite the relatively strong sequence conservation between the endonuclease domains of the two proteins (Additional file 5: Figure S5) [36,52]. This feature is useful for studies involving mouse cells and human ORF2 protein. We also ascertained that the epitope recognized by the monoclonal anti-ORF2p antibody includes amino acid 205 of the human ORF2p endonuclease domain. This amino acid is required for the ORF2p endonuclease activity and is therefore necessary for L1-driven retrotransposition (Figures 3 and 4). As a result, our antibody exhibits some bias toward detection of the ORF2 proteins containing a functional endonuclease domain at least relative to the status of amino acid 205. Additionally, an alignment of the consensus L1PA1-PA8 ORF2p sequences [52] demonstrated that L1PA3 to 5 have the same sequence as L1PA1, whereas L1PA2, 6, and 7 have one substitution in the core region surrounding amino acid 205 (200-210aa) (Additional file 5: Figure S5). The same analysis identified that L1PA8 varies by two amino acids from the L1PA1 sequence.

This discovery opens up the possibility that our monoclonal antibody may inhibit human L1 endonuclease activity. Suppression of L1 retrotransposition by HIV reverse transcriptase inhibitors has been previously reported [53,54], generating an interest in developing L1-specific inhibitors with the potential to suppress L1-associated damage *in vivo*. While the use of such RT inhibitors serves as a helpful tool to study the L1 replication cycle, these inhibitors are not specific to L1 as they are also expected to suppress telomerase RT [55]. Furthermore, they have significant side effects in humans [56] and it is not known whether the inhibition of the RT also prevents damage from the L1 endonuclease-induced DNA double-stranded breaks. Thus, inhibition of L1 endonuclease activity is an attractive approach in order to suppress most, if not all, of the L1-induced damage. The development of either chemical or antibody-based inhibitors are the two of the main approaches generally used for the suppression of enzymatic activities. In addition to the effective inhibition of enzyme activity, efficient delivery, stability, and lack of toxicity are common goals for both types of inhibitors [57,58]. The specificity of inhibition is a potential challenge with the development of L1 endonuclease inhibitors because this endonuclease is related to the human APE1, which is involved in the repair of DNA damage by the base excision repair pathway [59]. Using a fluorescence-based *in vitro* cleavage assay, we demonstrated that our monoclonal anti-ORF2p antibody can reduce L1 endonuclease activity by about 25% without any inhibitory effect on the *in vitro* activity of the human APE1 (Additional file 4: Figure S4). While it is not known yet whether the antibody is able to inhibit L1 endonuclease activity in the context of the full-length ORF2 protein or in the cellular environment, these results provide the first proof of principle that an antibody specific to amino acid 205 of the L1 endonuclease can reduce the activity of the enzyme.

## Conclusions

Our data demonstrate that this anti-ORF2p monoclonal antibody will be a useful tool for studies involving human L1 because it is specific to human ORF2p. The anti-ORF2p monoclonal antibody detects ORF2 protein generated from the ORF2 expression plasmid as well as both codon-optimized and wild-type full-length L1 expression plasmids transiently transfected into human cells. Our data also establish a rationale for the development of antibody-based inhibitors of L1-induced damage.

## Methods

### Cells

FLP-In™-293 (Invitrogen) cells were cultured in HyClone Dulbecco’s modified Eagle’s medium with 10% fetal bovine serum (Invitrogen) and maintained under 6% CO_2_ at 37°C. HeLa (ATCC CCL2), NIH-3T3 (ATCC CRL-1658), and Ntera2 (ATCC CRL-1973) cells were maintained as previously described [50].

### Transfections

Western blot: 293 cells were seeded at 1.5 × 10^6^ cells per T25 flask and transfected 16 to 18 hours later with 2 μg of the human or mouse ORF2 or EN expression plasmids [28], or 1, 2, or 4 μg of codon-optimized L1Pa1 [36] (L1co) or wild-type L1.3 (L1wt) [6,36]. Plus reagent (6 μL) and Lipofectamine (8 μL) (Invitrogen) were used for each ORF2 or EN transfection reaction in serum-free media; 12 μL of Plus reagent and 24 μL of Lipofectamine were used for each transfection reaction with L1co or L1wt in serum-free media. Transfections with maximum amount of the empty pCDNA plasmid were used as controls. After 3 hours, serum-free media was replaced with serum-containing media, and the cells were harvested at 24 hours after transfection unless otherwise noted in the figure. HeLa and NIH-3T3 cells were seeded at 2 × 10^6^ and 2.5 × 10^6^ cells per T75 flask, respectively, and transfected as previously described using 6 μg of plasmid [51], 12 μL of Plus reagent, and 18 μL or 24 μL of Lipofectamine, respectively, in serum-free media.

### Total protein extraction

Total protein was extracted as previously described [28,51] using phosphate buffered saline (PBS; 137 mM NaCl (Sigma S9888), 2.7 mM KCl (Sigma P4505), 10 mM Na_2_HPO_4_ (Sigma S3264), 2 mM KH_2_PO_4_ (Sigma P9791), pH = 7.4), 5 mM ethylenediaminetetraacetic acid (Sigma ED), and 0.02% sodium azide (Sigma S2002). Lysis buffer was supplemented with phosphatase inhibitors 2 and 3 (Sigma P5726 and P0044, respectively) and Halt Protease inhibitors at 10 μL/mL each. The samples were subjected to two freeze (−80°C)/thaw (25°C) cycles. The samples were sonicated three times for 10 seconds at 12 watts RMS using a 3 mm wide QSonica Microson homogenizer with Microson ultra sonic disruptor XL2000 (Misonix). The protein concentration of each sample was determined using 595 nm wavelength OD values against a bovine serum albumin standard.

### Western blot analysis

A 10 to 20 μg of total protein were combined with 2× Laemmli buffer and 1.6 μL (14.3 M) β-mercaptoethanol and boiled for 5 minutes prior to fractionation on Tris Acetate 3-8% Midi gels, Bis Tris 4 to 12% Midi gels (Invitrogen), and transferred onto nitrocellulose membranes (iBlot System; Invitrogen). Membranes containing fractionated protein samples were blocked for 1 hour in PBS-Tween containing 5% milk and incubated with a 1:250 dilution of custom polyclonal antibodies against the mouse ORF2p endonuclease [28], a 1:500 dilution of custom polyclonal antibodies against the human ORF2p endonuclease [27,28] antibodies, or a 1:250 dilution of custom monoclonal antibodies against the human ORF2p endonuclease overnight at 4°C. Detection was carried out using horseradish peroxidase (HRP)-conjugated secondary antibodies, either HRP-donkey anti-goat (Santa Cruz; sc-2020), HRP-donkey anti-rabbit (Santa Cruz; sc-2317), or HRP-goat anti-mouse (Santa Cruz; sc-2031) at a 1:5,000 dilution in 3% milk in PBS-Tween for 1 hour. A 1:5000 dilution of GAPDH antibodies (Santa Cruz sc-25778) was used as an equal loading control. A HRP conjugated monoclonal antibody against the 6× HIS tag (Pierce MA1-21315-HRP) was used at a 1:2000 dilution. All Western blots were developed using Clarity™ Western ECL Substrate (Bio-Rad, Cat. #170-5061).

For SDS Tris Glycine gels (Figure 5; Additional file 4: Figure S4), 3 to 20 μg of total protein were combined with 2× Tris Glycine SDS sample buffer and 1.6 μL (14.3 M) β-mercaptoethanol and boiled for 5 minutes prior to fractionation on Tris Glycine 4% Mini gels with Tris Glycine SDS running buffer (Invitrogen) and transfer onto nitrocellulose membranes. Membranes containing fractionated proteins were blocked for 1 hour in PBS-Tween containing 5% milk at room temperature. The membranes were then incubated overnight at 4°C with 1 mL of Ab-containing hybridoma supernatant in a blocking mixture containing 4 mL of media collected from NIH-3T3 cells cultured for 24 hours and 15 mL of 3% milk in PBS-Tween. Detection was carried out using HRP-conjugated secondary antibodies HRP-goat anti-mouse (Santa Cruz; sc-2031) at a 1:5,000 dilution in 3% milk in PBS-Tween for 1 hour. All Western blots were developed using Clarity™ Western ECL Substrate (Bio-Rad, Cat. #170-5061).

For Tris Glycine Native gel (Additional file 2: Figure S2), 100 ng of bacterially purified human ORF2p endonuclease was combined with 2× Native Tris Glycine sample buffer along with 5% GelCode Blue Stain Reagent (Thermo Scientific, Prod # 24592) and fractionated on a Tris Glycine 4 to 12% gel with Tris Glycine Native running buffer (Invitrogen). Fractionated proteins were transferred onto a nitrocellulose membrane. Membranes containing fractionated proteins were blocked for 1 hour in PBS-Tween containing 5% milk at room temperature. The membranes were then incubated overnight at 4°C with 1 mL of Ab-containing hybridoma supernatant in a blocking mixture containing 4 mL of media collected from NIH-3T3 cells cultured for 24 hours and 15 mL of 3% milk in PBS-Tween. Detection was carried out using HRP-conjugated secondary antibodies HRP-goat anti-mouse (Santa Cruz; sc-2031) at a 1:5,000 dilution in 3% milk in PBS-Tween for 1 hour. All Western blots were developed using Clarity™ Western ECL Substrate (Bio-Rad, Cat. #170-5061).

### Plasmids

All endonuclease constructs used in this study have been previously described [28], as well as ORF2 constructs [28] and L1PA1 (codon-optimized full-length L1) [36]. ‘L1wt’ is JM101/L1.3 no tag [6].

### ORF2p endonuclease purification

A human ORF2 endonuclease was expressed in bacteria and the EN protein was purified as previously described [31-33].

### Monoclonal antibody production

hORF2p endonuclease was bacterially purified as previously described [31,32]. This purified human ORF2 endonuclease protein was used for immunization of 6 Balb/c mice to generate monoclonal anti-ORF2p antibodies following a standard immunization protocol. Briefly, three sequential immunizations (with 2 week intervals between the injections) with antigen, (purified ORF2p endonuclease diluted in saline) in complete Freund’s Adjuvant for the first injection and incomplete Freund’s Adjuvant for the second and third injection, injected intraperitoneally, were performed. The fourth and final immunization was done using the antigen in saline. Mice were bled and tested using ELISA to determine which mouse to use as the source of B-cells for hybridoma production. Electrofusion was performed between B-cells harvested from the spleen and myeloma cells to produce hybridomas. Resulting hybridoma clones were screened with indirect ELISA to identify positive clones. The final stock of antibody was obtained by protein-G affinity column purification. The antibodies were stored in a PBS with 0.02% W/V sodium azide storage solution. The affinity purified hORF2p monoclonal antibodies were used for subsequent testing.

### The LINE-1 EN cleavage assay

The LINE-1 EN was expressed and purified as described previously [31,32]. The LINE1 EN cleavage assay was performed using 200 nM purified LINE1 EN, 100 nM of a duplexed oligonucleotide containing LINE-1 EN target site. The reaction buffer contained 20 mM Hepes (pH 6.5), 150 mM NaCl, 1 mM MgCl_2_, 1 mM dithiothreotol (DTT), 1% dimethyl sulfoxide (DMSO), 0.1% triton, and 0.01% sodium azide.

The effect of the monoclonal anti-ORF2p antibody on LINE1 EN activity was tested using three concentrations: 100 nM, 150 nM, and 200 nM. The antibody was diluted into the above described reaction buffer just prior to use. The same was done for the anti-hORF1p antibody [51]. A buffer control was used for background subtraction, in which the same volume of buffer alone as the volume of buffer containing antibody was added to the reactions. The LINE-1 EN and APE1 EN cleavage reactions were carried out at 37°C for 30 minutes. The reactions were stopped by quenching on ice and the addition of stop solution: 1× Tris borate EDTA buffer, 80% formamide, 0.01 mM EDTA, and xylene cyanol. The samples were run on 18% denaturing acrylamide gels and were analyzed using the Typhoon imager (GE Lifesciences). Fluorescense intensity (FI) was measured using Image Quant software (GE Lifesciences) and graphed using Prism software (GraphPad software, LLC). The percent inhibition of each reaction was determined using the following equation: % Inh = 100 × (1 − (FI_Antibody_ − FI_BufferControl_)/(FI_L1/APE1 EN_ − FI_Buffer Control_)).

### The APE1 EN cleavage assay

The purified APE1 EN was purchased from New England Biolabs. The assay was performed using 0.01 and 0.1 units of enzyme and 200 nM of duplexed oligonucleotide containing an abasic site. The sequence of the oligonucleotide was based upon previously published work [59]. The reaction buffer contained 50 mM potassium acetate 20 mM tris-acetate, 10 mM magnesium acetate, 1 mM DTT, 1% DMSO, 0.1% triton, and 0.01% sodium azide.

### Annealing oligonucleotides

All oligonucleotides were purchased from Integrated DNA Technologies. The oligonucleotides used in the assays were annealed by adding equivalent amounts of each complimentary nucleotide in annealing buffer (50 mM Hepes (pH 7.5) and 100 mM NaCl). The samples were incubated in boiling water for 5 minutes and slow cooled for 1 hour in the dark. The sequence for the LINE1 EN oligonucleotides used in the assay are as follows: 5′/AlexaFluor488/CCTTTTTTTTTAACCGC3′ and 5′GCGGTTAAAAAAAAAGG3′. The sequence for the APE1 EN oligonucleotides used in the assay are as follows: 5′/AlexaFluor488/GCCCCC_GGGGACGTACGATATCCCGCTCC3′ (where “_” represents an abasic site) and 5′GGAGCGGGATATCGTACGTCCCCCGGGGGC3′.

### Alignment of human and mouse ORF2p endonuclease domains

Human L1PA family consensus sequences [52] and L1 Spa [60] ORF2 sequence were converted to amino acid sequences and aligned using DNASTAR MegAlign program through the Clustal V method utilizing a gap penalty of ‘10’ and a gap penalty length of ‘10’.

### Calculation of the number of protein molecules

The molecular weight of all proteins was calculated based on their amino acid composition using EditSeq software. The number of molecules detected by monoclonal anti-ORF2p antibody was calculated using the following formula:

$$ \mathbf{X}\;\mathbf{molecules}=\left[\mathbf{Mass}\;\left(\mathrm{g}\right)\;/\;\mathbf{Molecular}\;\mathbf{weight}\;\mathbf{of}\;\mathbf{a}\;\mathbf{specific}\;\mathbf{protein}\;\left(\mathrm{g}\;/\;\mathrm{mol}\right)\right]\times \mathbf{6.022}\times \mathbf{1}{\mathbf{0}}^{23}\left({\mathrm{mol}}^{\hbox{-} 1}\right) $$

## Additional files

Additional file 1: Figure S1. Analysis of expression of functional and non-functional human ORF2 endonuclease domains in NIH-3T3 cells. Western blot analysis of proteins generated from expression plasmids containing codon-optimized functional (EN) and non-functional (EN 205,230) ORF2 endonuclease sequences transiently transfected in NIH-3T3 cells with anti-human ORF2p monoclonal antibody **(top)**, anti-human ORF2p polyclonal antibodies **(middle)**, or GAPDH **(bottom)**. Control lane indicates cells transiently transfected with an empty vector; 25 and 37 kDa are molecular markers. Additional file 2: Figure S2. Analysis of endogenous ORF2p in different cell lines. **(Top)** Western blot analysis of total cell lysate from the following cell lines: NIH-3T3, 293, Ntera2, and HeLa using hybridoma supernatant. Protein lysate from 293 cells transiently transfected with an expression plasmid containing codon-optimized ORF2 was used as a positive control for ORF2p expression (third lane). Control lane indicates 293 cells transiently transfected with an empty vector. Positions of molecular markers are indicated on the right as 100 or 150 kDa. **(Bottom)** The same experiment and analysis as in **(top)**, but using secondary antibodies only. Positions of molecular markers are indicated on the right as 100 or 150 kDa. Total amount of cell lysate loaded is in micrograms (μg). Additional file 3: Figure S3. Analysis of functional ORF2p endonuclease in native conformation. Western blot analysis of the bacterially purified ORF2p endonuclease (EN) fractionated under native conditions using hybridoma supernatant. Buffer lane indicates storage buffer used for purified ORF2p endonuclease. Additional file 4: Figure S4. Monoclonal anti-ORF2p antibody does not inhibit the APE1 endonuclease activity *in vitro*. **(Top)** SDS-PAGE analysis of *in vitro* APE1 endonuclease cleavage assay using monoclonal anti-ORF2 antibody. ORF2 antibody denotes the addition of 200 nM of the monoclonal anti-ORF2p antibody, control indicates the addition of the same volume of the buffer used for the reactions containing monoclonal anti-ORF2 antibody, and APE1 denotes bacterially purified human APE1 endonuclease; 0.1 and 0.01 units of APE1 were tested. **(Bottom)** Quantitation of the results of the *in vitro* APE1 endonuclease cleavage assay in A. Results were normalized to 0 nM control (n = 3). Equation used to determine percent (%) inhibition is listed in the methods section. Additional file 5: Figure S5. Analysis of ORF2p endonuclease conservation in human and mouse. Alignment of ORF2p endonucleases of L1Pa families in humans and the ORF2p endonuclease domain of mouse L1 Spa. Black arrow indicates area of the epitope of anti-ORF2p monoclonal antibody.

## Abbreviations

APE: Apurinic/apyrimidinic endonuclease 1
EN: N-terminal endonuclease
HRP: Horseradish peroxidase
kDa: Kilodalton
L1: Long interspersed element-1
ORFs: Open reading frames
PBS: Phosphate buffered saline
RNP: Ribonucleoprotein
RT: Reverse transcriptase
SINE: Short interspersed element
SVA: SINE-VNTR-Alu elements
