# Supplementary figures and images for: Development of a monoclonal antibody specific to the endonuclease domain of the human LINE-1 ORF2 protein
Source: Mob DNA. 2014 Dec 10;5:29. doi: 10.1186/s13100-014-0029-x (PMC4279459; doi:10.1186/s13100-014-0029-x)

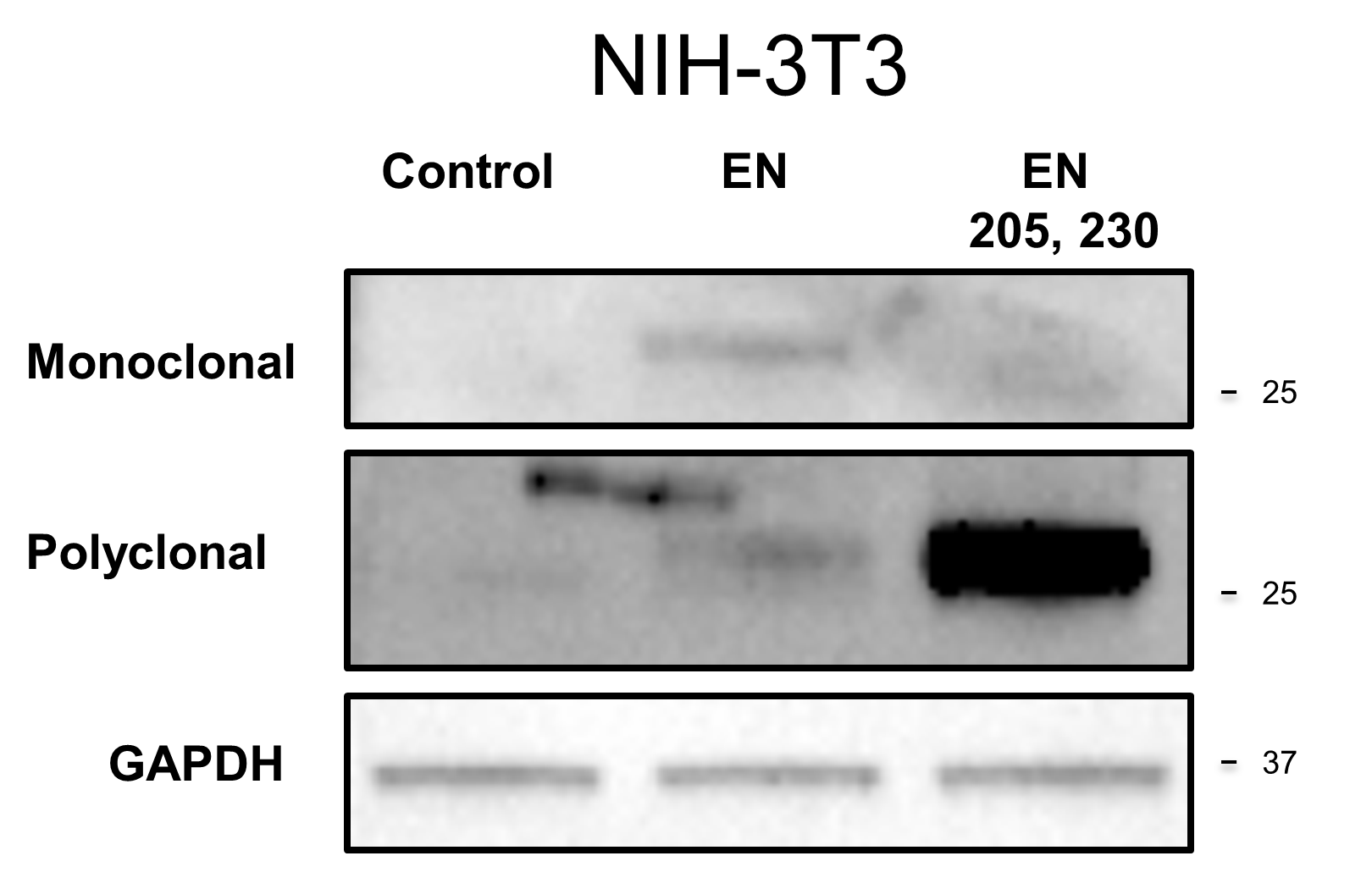

Supplement: Additional file 1: Figure S1. — Analysis of expression of functional and non-functional human ORF2 endonuclease domains in NIH-3T3 cells. Western blot analysis of proteins generated from expression plasmids containing codon-optimized functional (EN) and non-functional (EN 205,230) ORF2 endonuclease sequences transiently transfected in NIH-3T3 cells with anti-human ORF2p monoclonal antibody (top), anti-human ORF2p polyclonal antibodies (middle), or GAPDH (bottom). Control lane indicates cells transiently transfected with an empty vector; 25 and 37 kDa are molecular markers. [file 13100_2014_29_MOESM1_ESM.tiff]

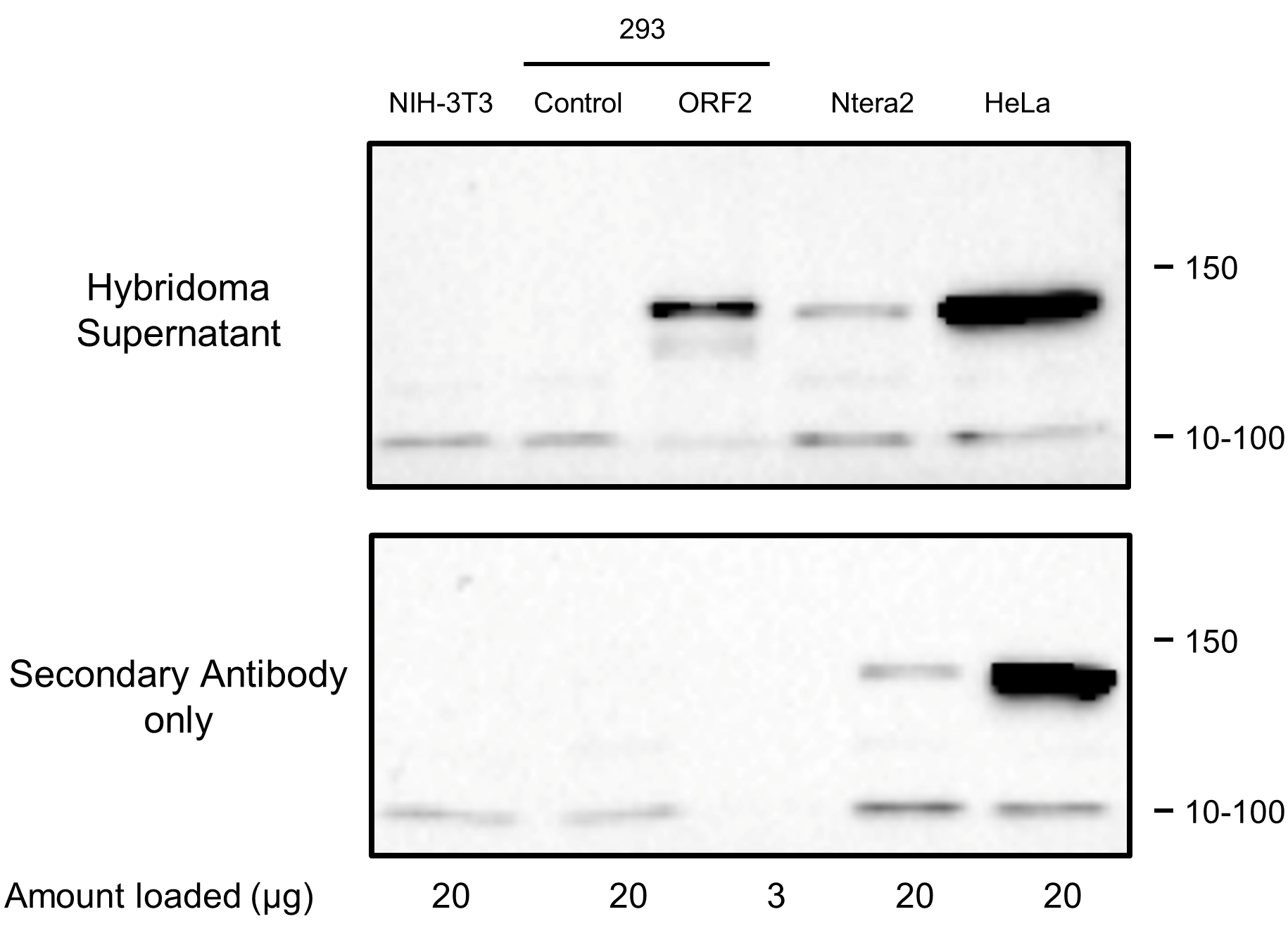

Supplement: Additional file 2: Figure S2. — Analysis of endogenous ORF2p in different cell lines. (Top) Western blot analysis of total cell lysate from the following cell lines: NIH-3T3, 293, Ntera2, and HeLa using hybridoma supernatant. Protein lysate from 293 cells transiently transfected with an expression plasmid containing codon-optimized ORF2 was used as a positive control for ORF2p expression (third lane). Control lane indicates 293 cells transiently transfected with an empty vector. Positions of molecular markers are indicated on the right as 100 or 150 kDa. (Bottom) The same experiment and analysis as in (top), but using secondary antibodies only. Positions of molecular markers are indicated on the right as 100 or 150 kDa. Total amount of cell lysate loaded is in micrograms (μg). [file 13100_2014_29_MOESM2_ESM.tiff]

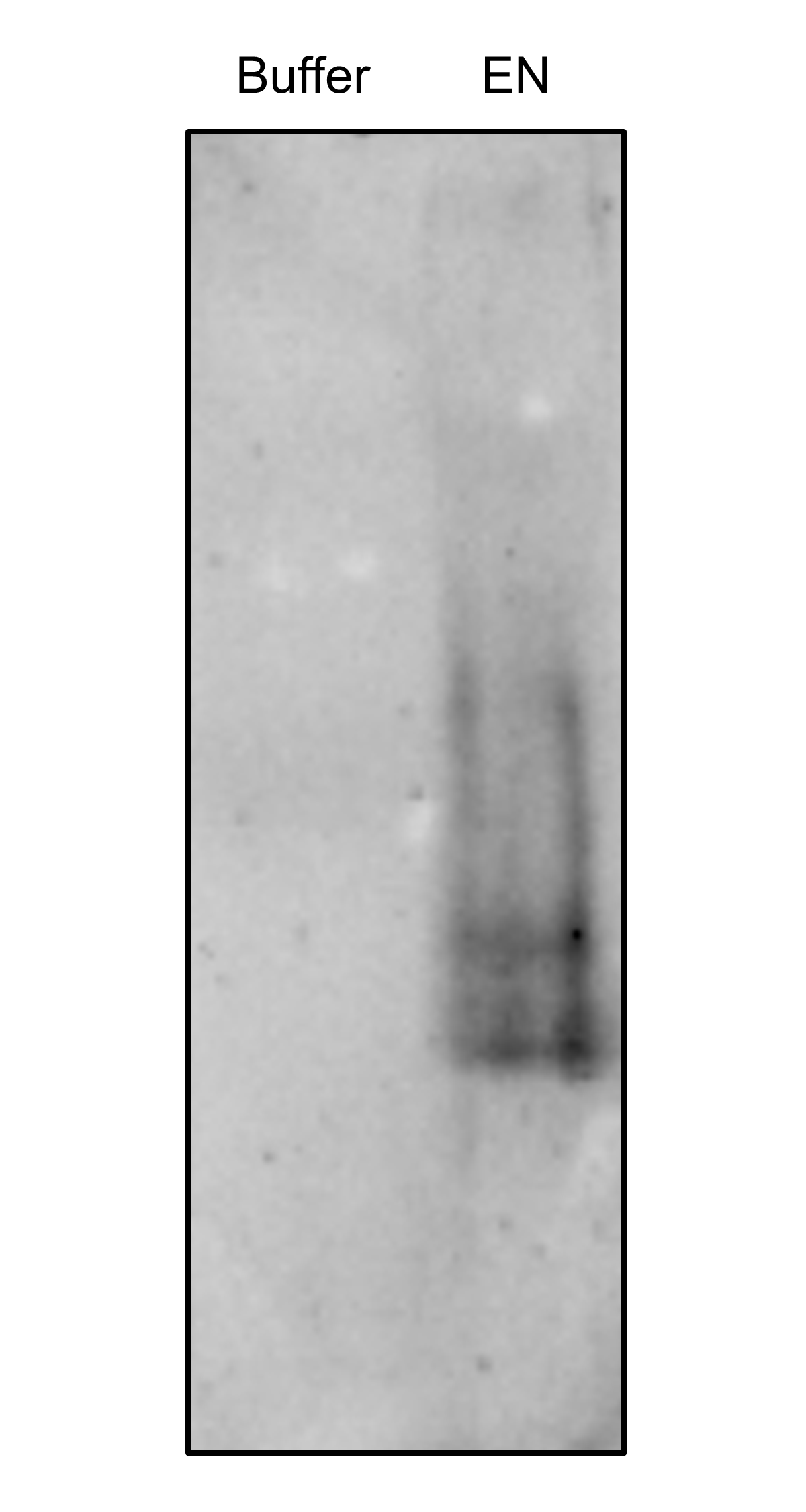

Supplement: Additional file 3: Figure S3. — Analysis of functional ORF2p endonuclease in native conformation. Western blot analysis of the bacterially purified ORF2p endonuclease (EN) fractionated under native conditions using hybridoma supernatant. Buffer lane indicates storage buffer used for purified ORF2p endonuclease. [file 13100_2014_29_MOESM3_ESM.tiff]

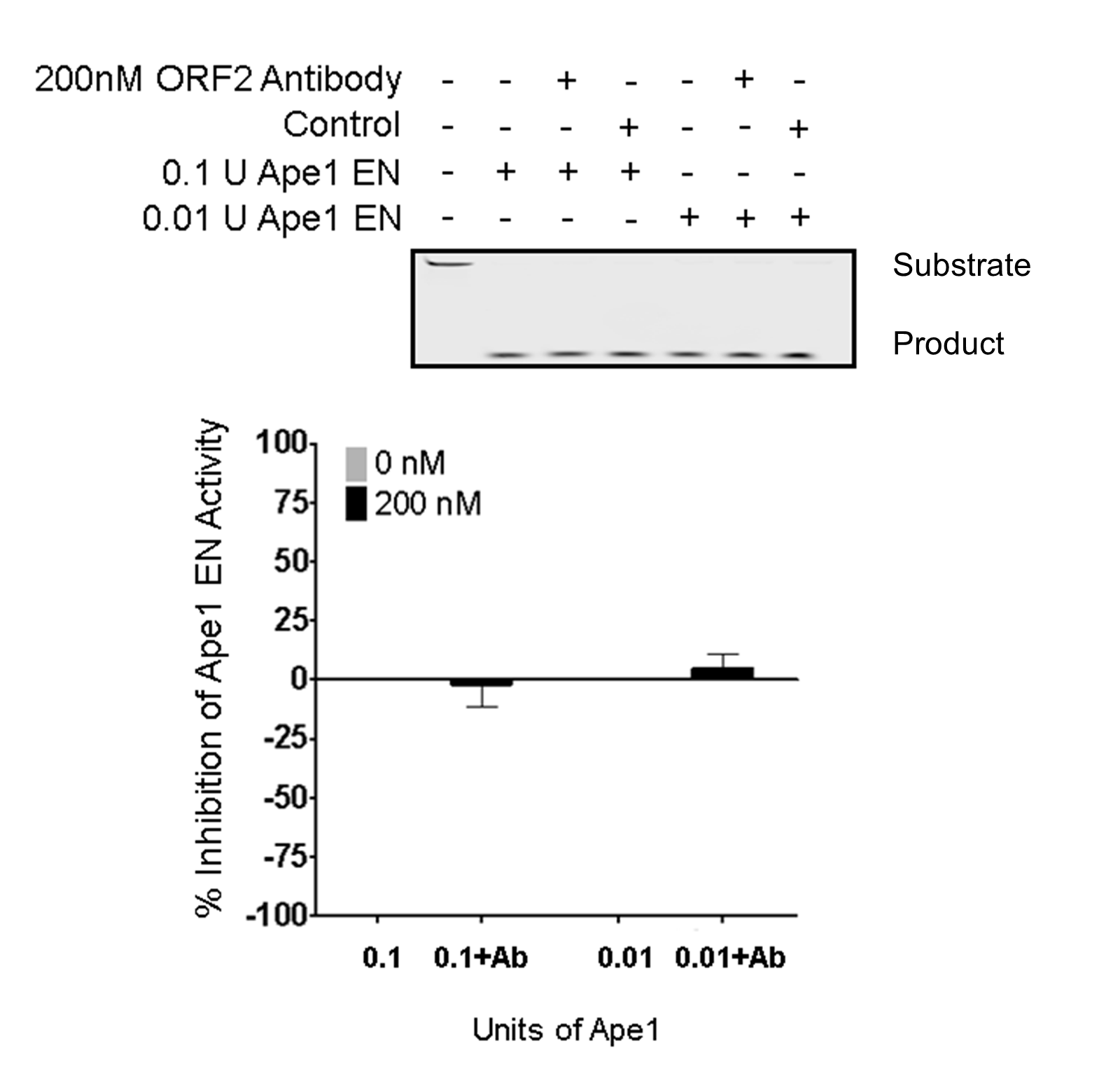

Supplement: Additional file 4: Figure S4. — Monoclonal anti-ORF2p antibody does not inhibit the APE1 endonuclease activity in vitro. (Top) SDS-PAGE analysis of in vitro APE1 endonuclease cleavage assay using monoclonal anti-ORF2 antibody. ORF2 antibody denotes the addition of 200 nM of the monoclonal anti-ORF2p antibody, control indicates the addition of the same volume of the buffer used for the reactions containing monoclonal anti-ORF2 antibody, and APE1 denotes bacterially purified human APE1 endonuclease; 0.1 and 0.01 units of APE1 were tested. (Bottom) Quantitation of the results of the in vitro APE1 endonuclease cleavage assay in A. Results were normalized to 0 nM control (n = 3). Equation used to determine percent (%) inhibition is listed in the methods section. [file 13100_2014_29_MOESM4_ESM.tiff]

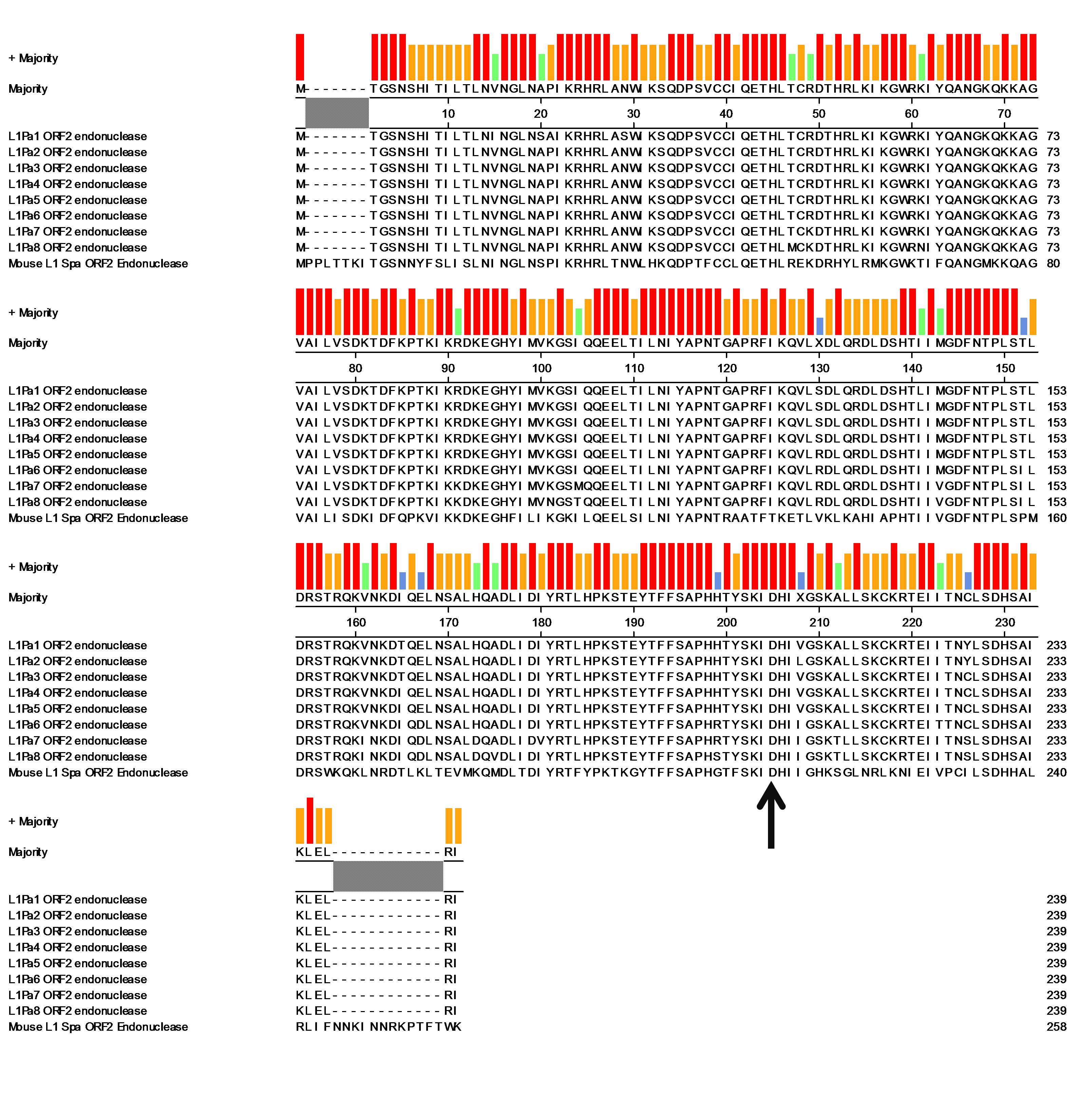

Supplement: Additional file 5: Figure S5. — Analysis of ORF2p endonuclease conservation in human and mouse. Alignment of ORF2p endonucleases of L1Pa families in humans and the ORF2p endonuclease domain of mouse L1 Spa. Black arrow indicates area of the epitope of anti-ORF2p monoclonal antibody. [file 13100_2014_29_MOESM5_ESM.tiff]
